# On the Medical Institutions of Cairo

**Published:** 1838-10

**Authors:** 


ON THE MEDICAL INSTITUTIONS OF CAIRO. BY CLOT BEY.

The hospital and school of medicine formerly existing at Abou-zabel have lately been removed to Cairo. Here also are formed an establishment for pharmacy, a new botanical garden, a museum for natural history, a civil hospital, and a "maternité." The removal of the hospital and school was rendered necessary by the distance at which it was formerly situated from Cairo. A large sum of money had been spent on the former establishment. It was required to find in Cairo a building sufficiently large to contain from one thousand to fifteen hundred patients, accommodation for three hundred students, and rooms appropriate for their instruction. The great college of Khasz and Hein, situated between old Cairo and Boulac, and opposite the island of Rhoda, at a little distance from the capital, has been chosen for the above purpose. The building is surrounded by fine walks. Its form is square: it has two stories above the ground-floor. All the wings consist of a double row of wards, separated by a corridor. Each wing is divided into four wards, each ward containing fifty beds. The first floor consists of vaulted chambers, which are used as stores. In the centre of the building is a large court, planted with trees. Connected with the south wing are four large buildings, separated one from the other. The first is employed for amphitheatres, chemical laboratories, cabinets of physics and natural history; the second for dormitories and refectories; the third for pharmacy; the fourth for kitchens, baths, and waiting places. At Abou-zabel there was a very excellent botanical garden. A portion of the island of Rhoda, so notorious for its gardens, and which rival the finest in Europe, is to be devoted to botanical purposes; Ibrahim Pacha being very much attached to agriculture, and therefore anxious to encourage botany. Already, under the direction of M. Regis, the young Arabians have made rapid progress in the study of natural history, and Egypt will, not long hence, be possessed of this science to a degree not at present at all suspected. The specimens contained in the cabinet of natural history have been collected from Egypt, Syria, Candia, Ardjas, Yemen, &c.; and applications have been made in France, England, Russia, Germany, Italy, &c. for an interchange of specimens, which has been the means of enlarging the collection. A building termed the Moristan was the miserable abode of insane patients, where they were chained in stone cells. Ibrahim Pacha had these unfortunate individuals removed to a civil hospital, where they are most assiduously cared for, better fed and better lodged. The necessity of a *maternité* has been strongly felt. The negresses and Abyssinian women have hitherto learned the art of midwifery in a school at Abou-zabel. Thirteen of them have already learned to write Arabic very correctly: they have studied the art of midwifery in a treatise translated into that language, together with demonstrations on an anatomical figure conducted by an European female teacher; and a professor to whom the teaching of the art is intrusted. A small hospital for females annexed to their school has afforded them an opportunity of superintending some deliveries; together with the practice of bleeding, vaccination, and surgical dressing. They have been instructed in some simple medical subjects, and in the preparation of some medicines. A distinguished student from the Maternité of Paris, (Madame Gault,) has been attached to the establishment as "accoucheuse en chef." She has found the female students very much advanced, and very capable of advancement. Their capacity for learning astonishes, particularly where it is considered how small a degree of intelligence is usually attributed to the negro race. The females here spoken of, are generally Abyssinians, and constitute a separate race; but among the negresses who study in the school, there is as much intelligence as among those of other races who are in the habit of considering them, in this respect, as beings of an inferior order. This is especially the case among the negresses of Sennar and of Meroni. There was therefore no obstacle to the formation of a midwifery hospital at Cairo. It is placed near the civil hospital. The females of the capital and of the provinces will be admitted, instructed, fed, and clothed, at the expense of the government. They will receive rewards in the same manner as the students of the school of medicine. Orphans, and the daughters of soldiers who have died or who are in active service, will be chiefly selected as students. The capital will furnish twenty students, and each province will supply four; making upwards of a hundred. A body of educated midwives will thus be formed, who will replace the ignorant and superstitious women to whom the duties of midwifery now devolve. The following is an instance of their ignorance and superstition. A poor woman had been three days in labour. Epithems, pessaries, the most absurd and dangerous applications had been employed: amulets had been tried, when a midwife suggested the efficacious means *of making a child dance between the legs of the patient,* in order to excite the child in the womb of the mother, and effect its delivery! These ignorant midwives are the cause of much evil both to the mothers and children. They possess secret remedies for curing barrenness: they have others, far more efficacious, for producing abortion; a crime which they commit without compunction. When a female does not wish to become a mother, the destruction of the fœtus is regarded by the midwives as a natural act, for which they are neither responsible to God or to society. They therefore persist without pity in this work of destruction. The discontinuance of this class of females will follow the formation of an educated class of midwives, who will fulfil another function of public utility, by treating the secret diseases to which females are subject, and for which they are prevented by a false modesty from applying to physicians. Prejudice is still so strong on this point, that a man would rather allow his daughter or his wife to die, than derogate from principles consecrated even in Arabian treatises of medicine. Fanaticism, by excluding females from society, has deprived them of the assistance of medicine, as well as of their paradise.

The above statement shows how much has been done in Egypt, in a little time. The great merit of all this belongs first of all to the viceroy; but his son, Ibrahim Pacha, has contributed very much to its accomplishment. The minister of public instruction has shown himself, in these matters, as in all others, worthy of the office which he has been called upon to fill.

*Gazette des Hopitaux. Janvier,* 1838.

